# Inhibition of Cancer Cell Growth by Exposure to a Specific Time-Varying Electromagnetic Field Involves T-Type Calcium Channels

**DOI:** 10.1371/journal.pone.0124136

**Published:** 2015-04-14

**Authors:** Carly A. Buckner, Alison L. Buckner, Stan A. Koren, Michael A. Persinger, Robert M. Lafrenie

**Affiliations:** 1 Departments of Biomolecular Sciences, Laurentian University, Sudbury, Ontario, Canada; 2 Regional Cancer Program, Health Sciences North, Sudbury, Ontario, Canada; 3 Behavioural Neurosciences, Laurentian University, Sudbury, Ontario, Canada; 4 Northern Ontario School of Medicine, Sudbury, Ontario, Canada; Dalhousie University, CANADA

## Abstract

Electromagnetic field (EMF) exposures affect many biological systems. The reproducibility of these effects is related to the intensity, duration, frequency, and pattern of the EMF. We have shown that exposure to a specific time-varying EMF can inhibit the growth of malignant cells. Thomas-EMF is a low-intensity, frequency-modulated (25-6 Hz) EMF pattern. Daily, 1 h, exposures to Thomas-EMF inhibited the growth of malignant cell lines including B16-BL6, MDA-MB-231, MCF-7, and HeLa cells but did not affect the growth of non-malignant cells. Thomas-EMF also inhibited B16-BL6 cell proliferation *in vivo*. B16-BL6 cells implanted in syngeneic C57b mice and exposed daily to Thomas-EMF produced smaller tumours than in sham-treated controls. *In vitro* studies showed that exposure of malignant cells to Thomas-EMF for > 15 min promoted Ca^2+^ influx which could be blocked by inhibitors of voltage-gated T-type Ca^2+^ channels. Blocking Ca^2+^ uptake also blocked Thomas-EMF-dependent inhibition of cell proliferation. Exposure to Thomas-EMF delayed cell cycle progression and altered cyclin expression consistent with the decrease in cell proliferation. Non-malignant cells did not show any EMF-dependent changes in Ca^2+^ influx or cell growth. These data confirm that exposure to a specific EMF pattern can affect cellular processes and that exposure to Thomas-EMF may provide a potential anti-cancer therapy.

## Introduction

Several studies have shown associations between electromagnetic field (EMF) exposure and health effects, such as cancer incidence; however, the conclusions of these studies are sometimes difficult to reproduce and are therefore controversial. It is difficult to make direct associations between EMF exposure and health effects, since not all EMFs are equivalent [1]. For example, the biological effects of exposure to the 50–60 Hz EMF pattern from electrical power lines cannot simply be compared to the effects of exposure to the megaHz patterns generated by cell phones.

While most studies have focussed on the negative effects of EMF, specific EMFs have been shown to accelerate wound healing, enhance musculoskeletal recovery, and disrupt tumor growth [2–4]. The mechanisms by which EMF affects biological processes are not well established. Some investigators have proposed that non-specific processes such as the generation of heat, formation of free-radicals, and promotion of DNA damage are involved [5–7]. However, the energies typically associated with low frequency EMF are not sufficient to cause changes in chemical bonds and other models including ion resonance have been proposed [8,9].

We hypothesize that the ability of EMFs to interact with biological processes is dependent on the temporal patterns of the fields, similar to the way pharmaceuticals are dependent on their structures [10]. Therefore, the information contained in a specific time-varying pattern, conveyed at low intensities (5–10 μT), could influence biological processes. The characteristics of an EMF that elicit biological responses should be specific for wave pattern, field strength, and exposure configuration.

Much attention has focused on the ability of low-frequency (<300 Hz) magnetic fields with simple or symmetrical (sine- or square-wave) patterns to affect cellular processes [11–13]. Some studies have shown that exposure to a low frequency EMF pattern can promote cell proliferation [5,14] while others have shown that EMF exposure inhibits cell proliferation [15–17]. Exposure of cells to 20–60 Hz EMF patterns has been shown to affect signal transduction pathways with effects on cAMP levels, MAP kinase activation, Ca^2+^-calmodulin kinase activation, or Ca^2+^ channels being the most commonly reported [11, 18–20].

Studies using asymmetrical EMF patterns, designed to mimic biologically-relevant processes, have shown that these complex EMF can influence specific biological processes [4, 21–23]. For example, the “Thomas” EMF pattern, a frequency modulated pattern designed to affect membrane activity associated with epileptic seizures, has been shown to have several biological effects. In particular, exposure of rodents to the Thomas-EMF pattern for 3 h/day has been associated with an increased analgesic response and to impaired memory performance on simple behavioral tasks [21, 24]. In these studies we showed that exposure to Thomas-EMF can also inhibit the growth of malignant cells by promoting Ca^2+^ uptake through T—type Ca^2+^ channels.

## Materials and Methods

### EMF exposure systems

**Fig 1:**
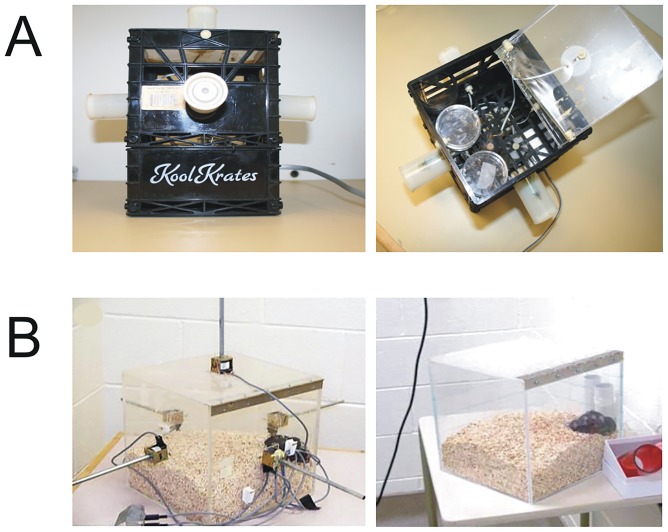
Diagram of the EMF exposure apparatus. A. *In vitro* exposure device is 12 cm on each side and fitted with 6 solenoids, one on each face of the cube, which are all activated during Thomas-EMF exposures. Plates containing cells are placed inside the cube for exposure. For the sham condition, the cells are placed in the box but electrical pulses are not sent to the solenoids. B. *In vivo* exposure device is 30 cm on each side and fitted with 6 solenoids, one on each face of the cube, and all solenoids are activated during Thomas-EMF exposure. The control box is not fitted with solenoids. A group of 5 mice is placed in the box for the exposure period.

**Fig 2:**
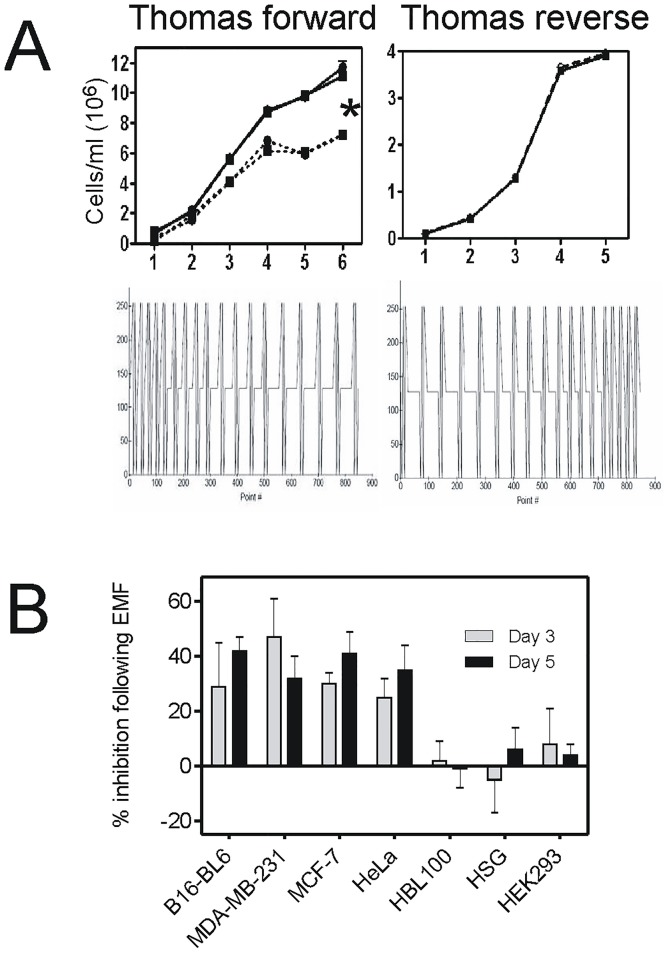
Exposure to Thomas-EMF alters cell proliferation. A. B16-BL6 cells were exposed to the Thomas-EMF pattern (dotted line) for 1 h/day (triangle) or 4 h/day (circle) or the Thomas-reverse-EMF for 1 h/day (dotted line) compared to a sham field (solid lines) and cell number determined on a hemocytometer. The data shows the average ± SD cell number for one of 3 experiments where * indicates p<0.05 between Thomas-EMF and sham groups (ANOVA). B. Malignant (MDA-MB-231, MCF-7, and HeLa) or non-malignant (HBL-100, HEK293T, and HSG) cell cultures were treated with the Thomas-EMF or sham field for 1 h/day and the average number of cells ± SD was determined each day. The percent inhibition in cell number for days 3 and 5 was determined [(sham—Thomas-treated)/sham] for at least 3 separate experiments and the average and standard deviation for each cell line is shown where * indicates p<0.05 between Thomas-EMF and sham groups.

The cultured cells or mice were exposed to Thomas-EMF, a weak 2–10 μT, frequency-modulated pattern, in a 4D box exposure system [21, 22] (Fig 1). The 4D box for the *in vitro* studies was 12 cm on each side, fitted with 6 wire-wound solenoids, and placed in a water-jacketed CO_2_ incubator at 37°C. The 4D box for *in vivo* exposures was made of plexiglass, 30 cm on each side, and fitted with six 50 ohm solenoids (28P-I-24VDC, Guardian Electric Manufacturing, Woodstock, IL, USA). The Thomas-EMF pattern was generated from a direct electrical current applied to the solenoids using a digital-to-analogue device controlled by the Complex© software on an IBM XT computer [21, 22]. Thomas-EMF is a digital file composed of 849 points each programmed for 3 msec (each cycle lasts 2.55 s) (Fig 2). It is composed of 18 doublet peaks (each singlet is 6 ms) with gradually increasing intervals; a 3 ms interval for the first 5 repeats (25Hz) to a 120 ms interval (6 Hz) for the last 5 repeats. The reverse Thomas-EMF shows the same pattern except that it proceeds from 6 Hz to 25 Hz.

### Cell lines

B16-BL6 (mouse melanoma)[25] and HSG (salivary gland)[26] cells [obtained from K.M. Yamada, NIH, Bethesda, MD], MDA-MB-231(breast cancer), MCF-7 (breast cancer), HeLa (cervical cancer), HBL-100 (breast) [27], and HEK293 (kidney) cells [ATCC, Manassas, VA] were maintained in Dulbecco’s Modified Essential Medium (DMEM, Hyclone, Logan, UT) containing 10% fetal bovine serum (Hyclone), 100 μg/ml streptomycin, and 100 U/ml penicillin (Invitrogen, Burlington, ON) at 37°C in 5% CO_2_.

### Cell proliferation

The cells were plated on day 0 at 20–30% confluence and exposed to Thomas-EMF or sham conditions for 1 h/day. Cell number was measured each day using a hemocytometer to count 8 fields for trypan blue-excluding cells and the mean ± SD determined for each of at least 3 experiments conducted on different days. For some experiments, the cells were treated with calcium channel activators or inhibitors for 30 min prior to exposure and media changed after exposure. Percent inhibition of cell proliferation was calculated as the difference in cell number between sham and Thomas-EMF treated cultures divided by the sham control [(sham—Thomas)/sham *100%].

### *In vivo* EMF exposure experiments in mice

A total of 35 male C57b mice (in 2 blocks of 25 and 10 animals conducted about 6 months apart) were used (Charles River, Pointe-Claire, QB). All procedures were approved by the Laurentian University Animal Care Committee in accordance with Canadian Council for Animal Care guidelines. The mice were housed in disposable plastic cages with 5 mice/cage, at 20–21°C, a 12:12 light:dark cycle, and fed a standard mouse diet and water *ad libitum*. For experiments, each mouse was injected subcutaneously in the right hind flank with 10^6^ B16-BL6 cells and randomly assigned to the sham or Thomas-EMF conditions. Mice were transferred to the 4D *in vivo* exposure system for 3 h each day, in the absence of food and water during treatment, starting the day after injection of the tumour cells and continuing until the day of sacrifice. Tumour growth was allowed to progress for 18–21 days for experiment 1 and 15–16 days for experiment 2 and then the tumours were excised and the diameter and weight measured. Tumour samples from the mice in experiment 2 were fixed in EFA (70% ethanol, 1.8% formaldehyde, and 0.75 N acetic acid), embedded in paraffin, and sectioned for immunohistochemical analysis [22]. The slides were stained using an ABC immunohistochemistry kit (Santa Cruz Biotechnology) and an antibody against Ki-67 [28]. TUNEL staining [29] was determined using an *In situ* Cell Death Detection kit (Roche Applied Science, Laval, QB). Staining intensity was assigned on a scale of 1–5 for each microscopic field and 5 fields/tumour were scored for each mouse and the average (and 95% CI) for 5 mice/condition measured.

### Acridine orange and ethidium bromide microscopy

B16-BL6 cell cultures were grown on glass coverslips in DMEM culture media and exposed to Thomas-EMF or sham conditions for 1 h/day for 1–5 days or to camptothecin for 6 h. The cells were incubated in 5 μg/ml acridine orange and 5 μg/ml ethidium bromide in PBS, pH 7.4, for 15 min and the green and red fluorescence visualized using an LSM510 fluorescence microscope.

### DNA fragmentation assay

B16-BL6 cells, cultured on 60x15 mm plates were treated with Thomas-EMF or sham for 1 h/day and harvested each day for 5 days. Cells treated with 6 μg/ml camptothecin for 6 or 24 h were used as a positive control. The cells were collected, lysed in TE-Triton buffer (0.2% Triton X-100, 10 mM Tris, 1 mM EDTA, pH 8.0) and subjected to centrifugation at 12,000xg for 15 min. The low molecular weight DNA-containing supernatant was treated sequentially with 50 μg/ml RNase A and 1 μg/ml proteinase K in 0.2% SDS and then precipitated using cold ethanol. The purified DNA was subjected to electrophoresis on a 1% agarose gel containing ethidium bromide.

### Fluorescence microscopic detection of calcium influx

Cells were cultured on 3 cm diameter glass coverslips and then labelled by incubation in 1 μM FLUO-4(AM) calcium indicator [30] for 15 min. For some experiments, the cells were then pre-treated with activators (Bay K8644) or inhibitors (bepridil, mibefradil, nifedipine, or verapamil) of voltage-dependent Ca^2+^ channels or suspending media 30 min prior to the calcium indicator. The coverslips were assembled in a Bioptechs FBS2 flow chamber on the microscope stage and exposed to sham or Thomas-EMF using a single solenoid. The background fluorescence associated with the cells was determined and images taken at 1, 15, 30, 45, and 60 min on a LSM510 fluorescence microscope with constant settings. The relative fluorescence was determined using MetaMax software and the fold increase ± SD for each time was reported for 3 independent experiments conducted on different days.

### Flow cytometry analysis

B16-BL6 cells were exposed to Thomas-EMF or sham conditions for 1 h/day, collected at 0–16 h post-exposure, and fixed in 70% ethanol at -20°C. The cells were pulsed for 4 h with 10 μM BrdU at 0–12 h following EMF exposure, processed using a 5-bromo-2'-deoxy uridine labelling kit from Roche (cat 11296736001), and stained with propidium iodide before analysis on a Beckman Coulter FC500 cytometer [31]. To detect cyclin expression, fixed cells were blocked in 1% FBS in PBS, pH 7.4, labelled in anti-cyclin (A, B, D, or E) antibodies (titre 1:20, Santa Cruz Biotech, Santa Cruz, CA), and stained with appropriate FITC- or rhodamine-labelled secondary antibodies (titre 1:100) [32]. The percent positive cells (mean ± SD) relative to secondary-antibody controls was determined for 3 experiments conducted on different days.

## Results

### Inhibition of cancer cell proliferation following exposure to Thomas-EMF

Daily exposure of B16-BL6 mouse melanoma cell cultures to Thomas-EMF inhibited their proliferation by 45±6% after 5 days compared to sham-exposed cells (Fig 2A). A 1 h/day exposure was sufficient since exposure for 4 h/day had the same effect. The specific time-modulated pattern of Thomas-EMF was critical since exposure to Thomas-reverse (ascending frequency modulation, 6Hz-25Hz) had no effect on B16-BL6 cell proliferation.

**Fig 3:**
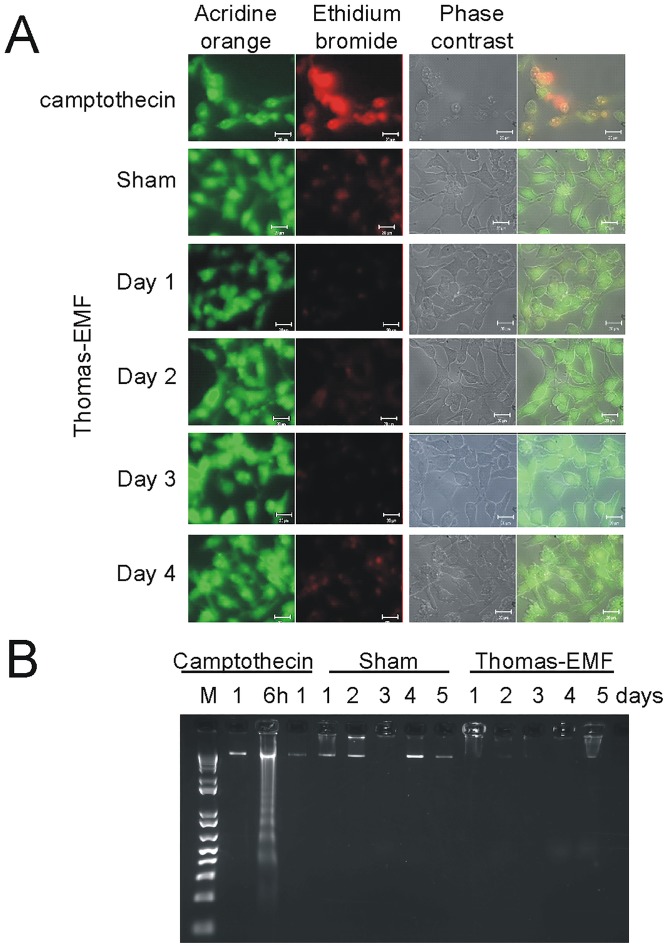
Exposure to Thomas-EMF does not promote apoptosis. A. B16-BL6 cells were treated with sham conditions, 10^-6^ M camptothecin for 6 h, or Thomas-EMF for 1 h/day for 1, 3, or 5 days. The cells were stained by incubation in 5 μg/ml acridine orange (green) and 5 μg/ml ethidium bromide (red) in PBS, pH 7.4 for 15 min and visualized on an LSM510 fluorescence microscope. B. B16-BL6 cells cultured in 3.5 cm culture dishes were treated with 10^-6^ M camptothecin for 1, 6, or 24 h (positive control) or exposed to Thomas-EMF or sham conditions for 1 h/day for 1–5 days. Cell were collected, lysed in TE/Triton buffer (0.2% TritonX-100, 10 mM Tris, 1 mM EDTA, pH 8.0), and subjected to centrifugation at 12 000 x g for 15 min. The low molecular weight DNA was treated with RNase and proteinase K and subjected to electrophoresis on an agarose gel containing ethidium bromide. M shows the 1kb DNA marker (Life Technologies).

Exposure of MDA-MB-231 and MCF-7 breast cancer cells and HeLa cervical cancer cells, to Thomas-EMF for 1 hour/day for 5 days also significantly (p<0.05) inhibited their proliferation by 30–50% compared to sham-exposed controls **(** Fig 2B). However, exposure of three non-malignant cell lines, HBL-100 breast cells, HEK293 kidney cells, and HSG salivary gland cells, to Thomas-EMF had no effect on cell proliferation. Exposure to Thomas-EMF did not promote cell apoptosis or death as determined by nuclear morphology and DNA fragmentation compared to the camptothecin-treated controls (Fig 3).

### Exposure to Thomas-EMF inhibited tumour growth in mice

**Fig 4:**
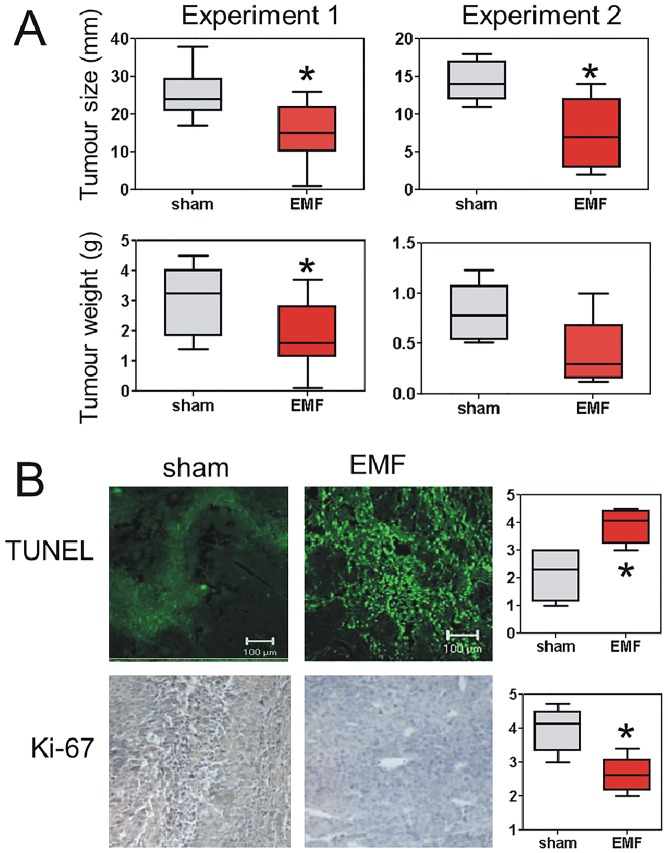
Exposure to Thomas-EMF inhibits B16-BL6 tumour growth in C57b mice. A. A total of 35 male C57b mice (experiment 1 = 25 and experiment 2 = 10 animals) were injected subcutaneously with 10^6^ B16-BL6 cells. The mice were randomly assigned to Thomas-EMF (red) or sham (grey) conditions and exposed for 3 h/day until the animals were sacrificed and tumours dissected. The average size and weight (and 95% CI and range) of the tumours are shown (* indicates p<0.05, Mann-Whitney U-test). B. The tumours from the mice in experiment 2 were fixed, embedded in paraffin, and sectioned for analysis for TUNEL and Ki-67 staining. Staining intensity for each section was assigned on a scale of 1–5 for each microscopic field. For analysis, 5 fields/tumour were scored for each mouse and the average (and 95% CI and range) of scores for 5 mice/condition were compared (* indicates p<0.05, Mann-Whitney U-test). Sections corresponding to average staining scores for each group are shown.

The effect of Thomas-EMF exposure on cancer cell growth *in vivo* was examined by measuring the growth of B16-BL6 cells implanted in C57b mice. The results of two studies showed that mice treated with 5–10 μT Thomas-EMF for 3 h/day had tumours that were 35–50% smaller in diameter and weight than mice exposed to sham conditions (p<0.05, Mann-Whitney U-test)**(** Fig 4A). Post-hoc power calculations showed that experiment 1 had >85% power to detect differences in both tumour weight and tumour size in response to EMF treatment with 95% confidence while experiment 2 had >85% power to detect a difference in tumour size but only a 70% power to detect a difference in tumour weight. The tumours from the EMF-treated mice were also noticeably less solid suggesting increased contributions from oedema.

Histochemical analysis of the tumours showed that TUNEL staining, which indicates DNA fragmentation [29], was higher in Thomas-EMF-treated mice compared to sham (p<0.05) suggesting an increase in tumour cell death (Fig 4B). Tumours isolated from the Thomas-EMF exposed mice also showed a lower level of proliferation, as indicated by decreased Ki-67 staining [28], compared to sham-exposed mice (p<0.05).

### Exposure to Thomas-EMF increased Ca^2+^ uptake

**Fig 5:**
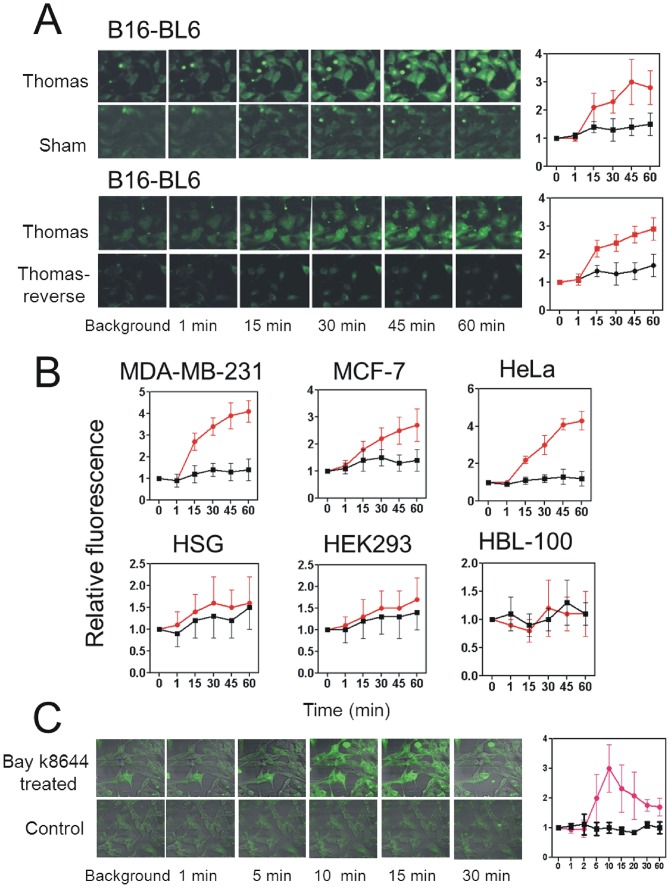
Exposure to Thomas-EMF enhances Ca^2+^ uptake in malignant cells. A. B16-BL6 cells were cultured on glass coverslips, labelled with the FLUO-4(AM) Ca^2+^ dye, placed on the microscope stage, and exposed to sham, Thomas-EMF or Thomas-reverse using a single solenoid. The background fluorescence was determined and images taken at 1, 15, 30, 45, and 60 min. The relative fluorescence of the entire microscopic field treated with Thomas-EMF (red) or sham field (black) was determined using MetaMax software and the mean ± SD fold increase for 3 independent experiments plotted as a function of duration of field exposure. B. The relative FLUO-4(AM)-dependent fluorescence was determined for MDA-MB-231, MCF-7, HeLa, HSG, HEK293, and HBL-100 cells treated with Thomas-EMF (red) or sham field (black). The graphs show the mean ± SD fold increase for 3 experiments determined using the MetaMax software as a function of exposure duration. C. B16-BL6 cells on glass coverslips were labelled with FLUO-4(AM) and mounted on the microcope stage. The background fluorescence was determined, the cells treated with 5 μM Bay K8644 and images taken at 1, 2, 5, 10, 15, 20, 30, 40, and 60 min. The relative fluorescence of the cells was determined and the mean ± SD fold change plotted as a function of duration of treatment.

Exposure of B16-BL6 cells to Thomas-EMF increased fluorescence of the Ca^2+^ dye, FLUO-4(AM) [30], within 15 min indicating an increase in Ca^2+^ influx compared to sham (Fig 5A and S1 Fig). This fluorescence increased 3.2±0.4 fold after a 1 h exposure and was observed in most cells within the field rather than in a subset. Consistent with the proliferation data, exposure of B16-BL6 cells to reverse-Thomas-EMF did not alter Ca^2+^ uptake and only malignant cells showed enhanced Ca^2+^ uptake following exposure to Thomas-EMF. FLUO-4(AM)-fluorescence was increased by 4.2 fold in HeLa, 2.8 fold in MCF-7, and 4.3 fold in MDA-MB-231 cells but was not altered in non-malignant HBL-100, HEK293, or HSG cells (Fig 5B).

Treatment of B16-BL6 cells with the voltage-dependent Ca^2+^ channel agonist, Bay K8644 [33], increased the fluorescence of the Ca^2+^ dye by 3.1±0.8 fold after 10 min of treatment indicating a rapid uptake of Ca^2+^ (Fig 5C). This is similar to the level of Ca^2+^ uptake promoted by Thomas-EMF treatment although Bay K8644 treatment induced a more rapid and transient increase in Ca^2+^.

**Fig 6:**
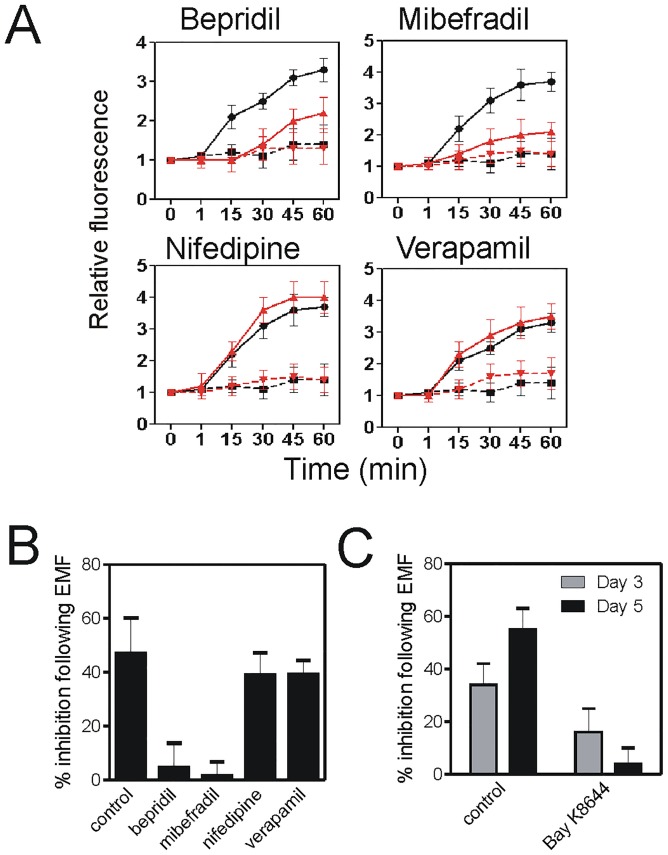
Thomas-EMF-dependent Ca^2+^ uptake and proliferation are mediated by T-type Ca^2+^ channels. A. B16-BL6 cells were treated with media (black lines) or inhibitors of L- or T-type Ca^2+^ channels for 30 min (red lines) and labelled with FLUO-4(AM). The cells were exposed to Thomas-EMF (solid lines) or sham field (dashed lines), and images taken at 1, 15, 30, 45, and 60 min. The relative fluorescence was determined using MetaMax software and the average ± SD fold increase for 3 independent experiments was plotted versus exposure time. B. The percent inhibition of B16-BL6 cells treated with Thomas-EMF for 5 days relative to sham-treated cells is shown in cells also treated with media (control), inhibitors of T-type Ca^2+^ channels (bepridil or mibefradil), or inhibitors of L-type channels (nifedipine or verapamil). The number of cells was determined for each condition and the percent inhibition (mean ±SD for 3 independent experiments) was determined as the difference between Thomas-EMF and sham-treated conditions relative to the control where * indicates p<0.05 (Students t-test). C. The percent inhibition of B16-BL6 cells treated with Bay K8644 or Thomas-EMF for 3 and 5 days relative to sham-treated cells is shown. The percent inhibition in cell proliferation (mean ± SD for 3 independent experiments) is shown.

Inhibitor studies showed that T-type voltage-dependent Ca^2+^ channels mediated Thomas-EMF-dependent Ca^2+^ uptake. Inhibitors of T-type Ca^2+^ channels (bepridil or mibefradil) significantly inhibited Ca^2+^ influx by 65–85% compared to sham while inhibitors of L-type voltage dependent Ca^2+^ channels (verapamil or nifedipine) had no effect (Fig 6A and S2 Fig).

The activation of the T-type Ca^2+^ channels also mediated the Thomas-EMF-dependent changes in cell proliferation. Exposure of B16-BL6 cells to Thomas-EMF inhibited cell proliferation by 48±7% after 5 days. Addition of the T-type Ca^2+^ channel blockers (bepridil or mibefradil), during the 1 h/day exposures, blocked the ability of Thomas EMF to decrease cell proliferation to <5% compared to sham (Fig 6B). In contrast, treatment with the L-type channel inhibitors had no significant effect on the ability of exposure to Thomas-EMF to inhibit cell proliferation (38–43% compared to sham). Treatment of B16-BL6 cells with Bay K8644 for 1 h/day did not have a significant effect on cell proliferation (<10% on days 3 and 5) compared to daily exposure to Thomas-EMF for 1 h/day (30–40% on days 3 and 5 of treatment) (Fig 6C).

### Exposure to Thomas-EMF alters cell cycle progression

**Fig 7:**
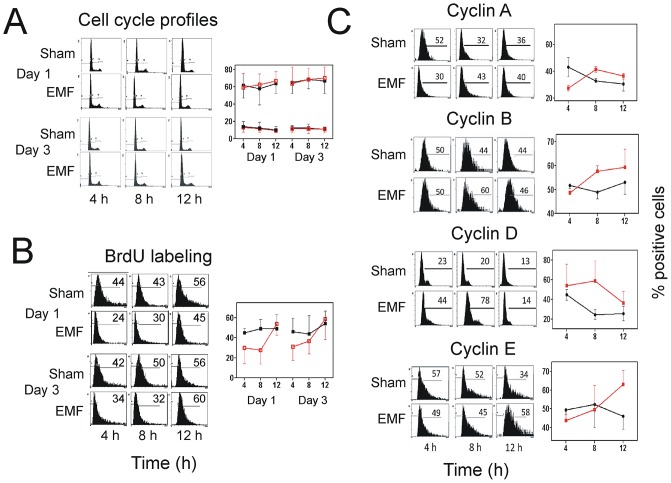
Exposure to Thomas-EMF alters cell cycle progression and cyclin expression. A. Unsynchronized B16-BL6 cells were exposed to Thomas-EMF (red lines) or sham conditions (black lines) for 1h, collected at times between 0 and 12 h post-exposure on days 1 and 3 of treatment. Cells were fixed, stained with propidium iodide, and analyzed on a Beckman Coulter FC600 flow cytometer (cell cycle profiles). The graph shows the average ± SD percentage of cells in the G1 phase (top) or G2/M phases (bottom) of the cell cycle for 3 independent experiments. B. B16-BL6 cells were exposed to Thomas-EMF (red lines) or sham conditions (black lines) for 1h, incubated with 10 μM BrdU for 4 h pulses after EMF exposure on day 1 and day 3 of treatment and fixed. The cells were processed and labelled using a 5-BrdU labelling kit from Roche (cat 11296736001) and analyzed on a Beckman Coulter FC600 flow cytometer (cell cycle profiles). The percent positive cells for a representative experiment are shown. The graph shows the average ± SD for the percentage of BrdU-staining cells for 3 independent experiments. C. B16-BL6 cells were exposed to Thomas-EMF (red lines) or sham (black lines) for 1 h and collected and fixed at 0–12 h post-exposure. Cyclin expression was detected using antibodies against cyclin A, B, D, or E, and stained with appropriate labelled secondary antibodies and analyzed on a Beckman Coulter FC600 flow cytometer. The percent positive cells for a representative experiment are shown. The graphs show the average ± SD for the percentage of positively stained cells for 3 independent experiments.

The ability of Thomas-EMF to inhibit cell proliferation without promoting cell death suggests altered cell cycle progression. Unsynchronized B16-BL6 cell cultures exposed to Thomas-EMF did not show differences in cell cycle profile (Fig 7A). However, incubation with BrdU, which incorporates into the DNA of dividing cells, showed that exposure to Thomas-EMF slowed entry into S phase (Fig 7B). In sham cells, BrdU staining was similar for all timepoints (44–50% positive) but Thomas-EMF-treated cells showed a significant decrease in staining for 4–8 h after exposure (24–34% positive) which was restored by 12 h post-exposure. Exposure to Thomas-EMF also promoted changes in cyclin expression. In B16-BL6 cells exposed to Thomas-EMF, the proportion of cells expressing cyclin A was decreased at 4 h post-exposure relative to the sham-treated control cells but was not different at 8 and 12 h post-exposure (Fig 7C). The proportion of cyclin B-expressing cells was increased at 8 and 12 h following Thomas-EMF treatment but was not altered in response to sham-treatment. The proportion of cells expressing cyclin D was significantly elevated 8 h after Thomas-EMF exposure but returned to the same level as sham-treated B16-BL6 cells by 12 h post-exposure. The proportion of cyclin E-expressing cells was elevated 12 h after Thomas-EMF treatment but remained relatively constant in response to sham-treatment.

## Discussion

The results of these studies showed that exposure to a low intensity, time varying EMF, Thomas-EMF, for 1 h/day inhibited the proliferation of malignant cells by 30–50% over 5 days. Several studies have shown that exposure of cells to symmetrical (sine-wave), low intensity EMF can inhibit cell proliferation [14–17]. These studies have usually involved exposure to a specific frequency of EMF between 20 and 100Hz [17, 34]; studies using 50 and 60 Hz EMF are most common because they correlate to environmental exposures [35]. It has been proposed that these exposures decrease the growth of cell cultures by enhancing cellular apoptosis as a result of EMF-dependent increases in reactive oxygen species, rapid influx of Ca^2+^, or activation of specific signaling pathways [5–7, 36]. A variety of models to explain these changes in cellular responses have been proposed involving changes in temperature, flux density, or energy input [37–40]. Some studies have shown increased expression of HSP70, a marker of cellular stress responses, in response to EMF exposures [12, 41]. Exposure to EMF (or electrical fields) has been shown to compromise plasma membrane integrity to allow influx of Ca^2+^ or chemotherapeutics to enhance cell death [42, 43]. The Thomas-EMF pattern is quite different from a symmetrical EMF and the mechanism by which it affects cell proliferation also appears to be quite different from some of those proposed.

The Thomas-EMF used in our experiments is a complex time-modulated pattern (25–6 HZ) which changes frequency during exposure. We have proposed that this pattern is critical for its effects on cells [10]. For example, while exposure to Thomas-EMF was able to inhibit cell proliferation, exposure to Thomas-reverse (ascending frequency, 6Hz-25Hz) did not affect cell proliferation. Since the energy profiles of these exposures are identical, the effect of the EMF was not caused by a general increase in energy exposure and was more consistent with models of stochastic resonance [8, 9]. Further, our experiments showed that exposure to Thomas-EMF did not enhance cell death as measured by the absence of apoptotic cell morphology, a sub-G1 peak on flow cytometery, and DNA fragmentation.

We also examined several cell lines to determine the generalizability of the effect of Thomas-EMF. In this study, exposure to Thomas-EMF inhibited the proliferation of the four malignant cell lines tested but did not affect the proliferation of the three non-malignant cell lines. This suggests that Thomas-EMF specifically targets some property of malignant cells and suggests it may be useful as an anti-cancer agent. This was not completely surprising since exposure to other EMF patterns (including 50 and 60 Hz EMF) have been shown to differentiate between cell types, depending on malignancy or tissue of origin, in promoting inhibition of proliferation [7, 34, 44, 45]. Some experiments had shown that EMF could promote anti-proliferative effects in leukocytes only when the cells were activated by mitogens [46].

The ability of Thomas-EMF to inhibit the proliferation of B16-BL6 cells was also present *in vivo*. B16-BL6 cells implanted in syngeneic C57b mice and treated with Thomas-EMF for 3 h/day formed tumours that were 35–50% smaller and less solid than in sham-treated mice. Since the average weight and health of the animals was not affected it appeared that exposure to Thomas-EMF did not negatively impact non-malignant tissues. The level of cell proliferation in the tumour tissues, as measured by Ki-67 expression, was decreased in Thomas-EMF-exposed mice while the level of cell death, measured as DNA fragmentation using the TUNEL assay, was higher in Thomas-EMF-exposed mice. This is distinct from the *in vitro* data which showed no increase in DNA fragmentation and may relate to changes in leukocyte infiltration seen in Thomas-EMF-exposed mice [22]. Others have also shown that exposure to 60Hz EMF can elevate immune function in treated animals [47] and exposure to 7.5 Hz, 0.4 T EMF can inhibit B16-F10 mouse tumour proliferation while increasing T cell and dendritic cell numbers in spleen [48]. This suggests that the anti-cancer effects of EMF exposure may also have an immune component. Further, some experiments using more complex patterns, such as overlapping multiple frequencies [49] or patterns recorded from cancer cells [4, 23] exposure can inhibit cancer growth in animal models or human patients.

Exposure to Thomas-EMF promoted Ca^2+^ influx in B16-BL6, MDA-MB-231, MCF-7, and HeLa malignant cells within 15 min which was increased 3–4 fold after 60 min compared to sham-treated cells. Non-malignant HBL-100, HEK293, and HSG cells exposed to Thomas-EMF did not alter cytoplasmic Ca^2+^ levels. The observation that Thomas-EMF affected Ca^2+^ uptake only in the malignant cells is consistent with the effect on cell proliferation. In addition, exposure to Thomas-reverse also did not promote Ca^2+^ uptake consistent with its inability to affect cell proliferation. Treatment of B16-BL6 cells with Bay K8644, an agonist of voltage-dependent Ca^2+^ channels [33], also increased Ca^2+^ uptake by 3–4 fold, similar to exposure to Thomas-EMF suggesting these changes are typical for cells under these conditions. However, the kinetics of Ca^2+^ uptake were quite different: treatment with Bay K8644 promoted a maximal uptake in cytoplasmic Ca^2+^ within 10 min which declined to background by 30 min while exposure to Thomas-EMF promoted a gradual increase in Ca^2+^ which was maintained for over 60 min. A variety of studies have implicated Ca^2+^ fluxes via voltage-dependent Ca^2+^ channels in responses to EMF exposure [50]. For example, differentiation of neuronal cell lines in response to exposure to 50 or 60 Hz EMF was shown to be inhibited by treatment with nifedipine, a blocker of L-type Ca^2+^ channels [51, 52] and one study has shown that exposure of a mouse neuronal cell line to 50 Hz EMF can block Ca^2+^ uptake through T-type voltage-dependent Ca^2+^ channels [20].

In these experiments, we used inhibitor studies to show that Thomas-EMF promoted Ca^2+^ uptake via T-type voltage-dependent Ca^2+^ channels. Pre-treatment with bepridil and mibefradil, which are well-described inhibitors of T-type channels, significantly blocked Ca^2+^ uptake in response to Thomas-EMF while pre-treatment with nifedipine and verapamil, which are inhibitors of L-type channels, had no effect. This indicates that exposure to Thomas-EMF enhances Ca^2+^ uptake by affecting T-type Ca^2+^ channel permeability.

The Thomas-EMF-dependent effect on Ca^2+^ influx appeared to be responsible for effects on cell proliferation. Blocking Ca^2+^ influx through the T-type Ca^2+^ channels also blocked the ability of Thomas-EMF to inhibit cell proliferation. In B16-BL6 cells treated with bepridil or mibefradil, the Thomas-EMF and sham-treated cultures grew at the same rate. In contrast, treatment of cells with inhibitors of L-type Ca^2+^ channels still showed decreased proliferation when exposed to Thomas-EMF.

The observation that Thomas-EMF can only impact proliferation of malignant cells may be related to the observation that many malignant cell lines and tumours aberrantly express T-type Ca^2+^ channels while non-malignant cells and HEK293 cells do not [53, 54]. For example, MCF-7 and MDA-MB-231 breast cancer cells have been shown to express T-type Ca^2+^ channel subunits while non-malignant breast cells do not [55, 56]. It is not clear why cancer cells express T-type Ca^2+^ channels although it has been correlated with increased malignant behaviour [56, 57]. Thus, exposure to Thomas-EMF might affect any cell that expresses the T type Ca^2+^ channel, such as sensory neurons, and may explain why Thomas-EMF inhibits pain responses in animal experiments [21, 24, 58].

The idea that Thomas-EMF can slow cell proliferation via changes in cytoplasmic Ca^2+^ is supported by studies that show Ca^2+^ is linked to changes in cell cycle progression and cyclin expression [59, 60]. Thomas-EMF delayed S phase entry for up to 8 h after the B16-BL6 cells were exposed as shown by a decrease in BrdU incorporation and consistent with the 12 h delay in cyclin E expression [61] compared to sham controls. Further, exposure to Thomas-EMF also altered cyclins A, B, and D, expression suggesting a delay at the G2/M cell cycle transition. An elevation in cyclin D levels 8 h after exposure and in cyclin B levels [62] 8–12 h after exposure are consistent with a delay in cell cycle progression through G2. Thus, the changes in cyclin expression indicate that the Thomas-EMF treated cells are delayed by 4–8 h in passing through G1/S and M phases which could account for the decreased levels of cell proliferation seen in the treated cultures.

These observations are consistent with the idea that exposure to specific EMF patterns can affect biological systems by a mechanism consistent with molecular resonance. In this case, exposure to Thomas-EMF was able to alter T-type Ca2+ channel permeability to allow an inappropriate influx of Ca^2+^ which was able to disrupt proliferation of malignant cells. These observations suggest that the Thomas-EMF could provide a potential anti-cancer therapy.

## Supporting Information

S1 Fig Exposure to Thomas-EMF enhances Ca^2+^ uptake in malignant cells. (PPTX)

S2 Fig Thomas-EMF-dependent Ca^2+^ uptake and proliferation are mediated by T-type voltage-dependent Ca^2+^ channels. (PPTX)

## References

[1] Cifra M, Fields JZ, Farhadi A. Electromagnetic cellular interactions. Prog Biophys Mol Biol. 2011; 105: 223–246. 10.1016/j.pbiomolbio.2010.07.003 20674588

[2] Assiotis A, Sachinis NP, Chalidis BE. Pulsed electromagnetic fields for the treatment of tibial delayed unions and nonunions. A prospective clinical study and review of the literature. J Orthop Surg Res. 2012; 7: 24 10.1186/1749-799X-7-24 22681718 PMC3441225

[3] Guo L, Kubat NJU, Isenberg RA. Pulsed radio frequency energy (PRFE) use in human medical applications. Electromagn Biol Med. 2011; 30: 21–45. 10.3109/15368378.2011.566775 21554100

[4] Costa FP, de Oliveira ASC, Meirelles R, Machado MC, Zanesco T, Surjan R, et al Treatment of advanced hepatocellular carcinoma with very low levels of amplitude-modulated electromagnetic fields. Br J Cancer. 2011; 105: 640–648. 10.1038/bjc.2011.292 21829195 PMC3188936

[5] Wolf FI, Torsello A, Tedesco B, Fasanella S, Boninsegna A, D’Ascenzo M, et al 50 Hz extremely low frequency electromagnetic fields enhance cell proliferation and DNA damage: Possible involvement of a redox mechanisms. Biochim Biophys Acta. 2005; 1743: 120–129. 15777847 10.1016/j.bbamcr.2004.09.005

[6] Vijayalaxmi, Prihoda TJ. Genetic damage in mammalian somatic cells exposed to extremely low frequency electro-magnetic fields: a meta-analysis of 97 publications (1990–2007). Int J Radiat Biol. 2009; 85: 196–213. 10.1080/09553000902748575 19296340

[7] Simko M. Cell type specific redox status is responsible for diverse electromagnetic field effects. Cur Med Chem. 2007; 14: 1142–1152. 10.2174/092986707780362835 17456027

[8] Blank M, Goodman R. Electromagnetic fields stress living cells. Pathophysiol. 2009; 16: 71–78. 10.1016/j.pathophys.2009.01.006 19268550

[9] Blanchard JP, Blackman CF. Clarification and application of an ion parametric resonance model for magnetic field interactions with biological systems. Bioelectromagnetics. 1994; 15: 217–238. 8074738 10.1002/bem.2250150306

[10] Whissell PD, Persinger MA. Emerging synergisms between drugs and physiologically-patterned weak magnetic fields: implications for neuropharmacology and the human population in the twenty-first century. Curr Neuropharmacol. 2007; 5: 278–288. 10.2174/157015907782793603 19305744 PMC2644491

[11] Nie K, Henderson A. MAP kinase activation in cells exposed to a 60 Hz magnetic field. J Cell Biochem. 2003; 90: 1197–1206. 14635193 10.1002/jcb.10704

[12] Lin H, Blank M, Rossol-Haseroth K, Goodman R. Regulating genes with electromagnetic response elements. J Cell Biochem. 2001; 81: 143–148. 11180404 10.1002/1097-4644(20010401)81:1<143::aid-jcb1030>3.0.co;2-4

[13] Pilla A, Fitzsimmons R, Muehsam D, Wu J, Rohde C, Casper D. Electromagnetic fields as first messenger in biological signaling: Application to calmodulin-dependent signaling in tissue repair. Biochim Biophys Acta. 2011; 10: 1236–1245. 10.1016/j.bbagen.2011.10.001 22005645

[14] Li X, Zhang M, Bai L, Bai W. Effects of 50 Hz pulsed electromagnetic fields on the growth and cell cycle arrest of mesenchymal stem cells: an *in vitro* study. Electromagn Biol Med. 2012; 31: 356–364. 10.3109/15368378.2012.662194 22676915

[15] Schimmelpfeng J, Dertinger H. Action of a 50 Hz magnetic field on proliferation of cells in culture. Bioelectromagnetics. 1997; 18 177–183. 9084869 10.1002/(sici)1521-186x(1997)18:2<177::aid-bem11>3.0.co;2-o

[16] Lange S, Viergutz T, Simko M. Modifications in cell cycle kinetics and in expression of G1 phase-regulating proteins in human amniotic cells after exposure to electromagnetic fields and ionizing radiation. Cell Prolif. 2004; 37: 337–349. 15377333 10.1111/j.1365-2184.2004.00317.x PMC6496295

[17] Yan J, Dong L, Zhang B, Qi N. Effects of extremely low-frequency magnetic field on growth and differentiation of human mesenchymal stem cells. Electromagn Biol Med. 2010; 29: 165–176. 10.3109/01676830.2010.505490 20923323

[18] Thumm S, Löschinger M, Glock S, Hämmerle H, Rodemann HP. Induction of cAMP-dependent protein kinase A activity in human skin fibroblasts and rat osteoblasts by extremely low-frequency electromagnetic fields. Radiat Environ Biophys. 1999; 38: 195–199. 10525956 10.1007/s004110050155

[19] Schimmelpfeng J, Stein JC, Dertinger H. Action of 50 Hz magnetic fields on cyclic AMP and intercellular communication in monolayers and spheroids of mammalian cells. Bioelectromagnetics. 1995; 16: 381–386. 8789069 10.1002/bem.2250160606

[20] Cui Y, Liu X, Yang T, Mei YA, Hu C. Exposure to extremely low-frequency electromagnetic fields inhibits T-type calcium channels via AA/LTE4 signaling pathway. Cell Calcium. 2014; 55: 48–58. 10.1016/j.ceca.2013.11.002 24360572

[21] Martin LJ, Koren SA, Persinger MA. Thermal analgesis effects from weak, complex magnetic fields and pharmacological interactions. Pharmacol Biochem Behav. 2004; 78: 217–227. 15219761 10.1016/j.pbb.2004.03.016

[22] Hu JH, St-Pierre LS, Buckner CA, Lafrenie RM, Persinger MA. Growth of injected melanoma cells is suppressed by whole body exposure to specific spatial-temporal configurations of weak intensity magnetic fields. Int J Radiat Biol. 2010; 86: 79–88. 10.3109/09553000903419932 20148694

[23] Zimmerman JW, Pennison MJ, Brezovich I, Yi N, Yang CT, Ramaker R, et al Cancer cell proliferation is inhibited by specific modulation frequencies. Br J Cancer. 2012; 106: 307–313. 10.1038/bjc.2011.523 22134506 PMC3261663

[24] Shupak NM, Prato FS, Thomas AW. Human exposure to a specific pulsed magnetic field: effects on thermal sensory and pain thresholds. Neurosci Lett. 2004; 363: 157–162. 15172106 10.1016/j.neulet.2004.03.069

[25] Fidler IJ (1975) Biological behavior of malignant melanoma cells correlated to their survival *in vivo* . Cancer Res 35: 218–224. 1109790

[26] Lam K, Zhang L, Bewick M, Lafrenie RM. HSG cells differentiated by culture on extracellular matrix involves induction of S-adenosylmethione decarboxylase and ornithine decarboxylase. J Cell Physiol. 2005; 203: 353–361. 15521072 10.1002/jcp.20247

[27] Gaffney EV. A cell line (HBL-100) established from human breast milk. Cell Tissue Res. 1982; 227: 563–568. 6891286 10.1007/BF00204786

[28] Gerdes J, Lemke H, Baisch H, Wacker HH, Schwab U, Stein H. Cell cycle analysis of a cell proliferation-associated human nuclear antigen defined by the monoclonal antibody Ki-67. J Immunol. 1984; 133: 1710–1715. 6206131

[29] Arends MJ, Morris RG, Wyllie AH. Apoptosis. The role of the endonuclease. Am J Pathol. 1990; 136: 593–608. 2156431 PMC1877493

[30] Gee KR, Brown KA, Chen WN, Bishop-Stewart J, Gray D, Johnson I. Chemical and physiological characterization of fluo-4 Ca^2+^-indicator dyes. Cell Calcium. 2000; 27: 97–106. 10756976 10.1054/ceca.1999.0095

[31] Guo B, Romero J, Kim BJ, Lee H. High levels of Cdc7 and Dbf4 proteins can arrest cell-cycle progression. Eur J Cell Biol. 2005; 84: 927–938. 16325502 10.1016/j.ejcb.2005.09.016

[32] Bianchi S, Fabiani S, Muratori M, Arnold A, Sakeguchi K, Miki T, et al Calcium modulates the cyclin D1 expression in a rat parathyroid cell line. Biochim Biophys Res Commun. 1994; 204: 691–700. 7980531 10.1006/bbrc.1994.2515

[33] Zahradnikova A, Minarovic I, Zahradnik I. Competitive and cooperative effects of Bay K8644 on the L-type calcium channel current inhibition by calcium channel antagonists. J Pharmacol Exp Therpeutics. 2007; 322: 638–645. 17475903 10.1124/jpet.107.122176

[34] Crochetti S, Beyer C, Schade G, Egli M, Frohlich J, Franco-Obregon A. Low intensity and frequency pulsed electromagnetic fields selectively impair breast cancer cell viability. PLOS ONE. 2013; 8: e72944 10.1371/journal.pone.0072944 24039828 PMC3770670

[35] Tomitsch J, Dechant E. Exposure to electromagnetic fields in households—trends from 2006 to 2012. Bioelectromagnetics. 2015; 36: 77–85. 10.1002/bem.21887 25421708

[36] Sadeghipour R, Ahmadian S, Bolouri B, Pazhang Y, Shafiezadeh M. Effects of extremely low-frequency pulsed electromagnetic fields on morphological and biochemical properties of human breast carcinoma cells (T47D). Electromagn Biol Med. 2012; 31: 425–435. 10.3109/15368378.2012.683844 22676212

[37] Weaver JC, Vaughan TE, Martin GT. Biological effects due to weak electric and magnetic fields: The temperature variation threshold. Biophysical J. 1999; 76: 3026–3030. 10354428 10.1016/S0006-3495(99)77455-2 PMC1300272

[38] Ushiyama A, Ohkubo C. Acute effects of low-frequency electromagnetic fields on leukocyte-endothelial interactions *in vivo* . In vivo. 2004; 18: 125–132. 15113039

[39] Brizhik L, Cruzeiro-Hansson L, Eremko A. Influence of electromagnetic radiation on molecular solitons. J Biological Phys. 1998; 24: 19–39. 10.1023/A:1005096714234 23345667 PMC3455866

[40] Panagopoulos DJ, Johansson O, Carlo GL. Evaluation of specific absorption rate as a dosimetric quantity for electromagnetic field effects. PLOS ONE. 2013; 8: e62663 10.1371/journal.pone.0062663 23750202 PMC3672148

[41] Tokalov SV, Gutzeit HO. Weak electromagnetic fields (50 Hz) elicit a stress response in human cells. Environ Res. 2004; 94: 145–151. 14757377 10.1016/s0013-9351(03)00088-4

[42] Stratton D, Lange S, Inal JM. Pulsed extremely low-frequency magnetic fields stimulate microvesicle release from human monocytic leukaemia cells. Biochem Bioph Res Commun. 2013; 430: 470–475. 10.1016/j.bbrc.2012.12.012 23237811

[43] Saliev T, Tachibana K, Bulanin D, Mikhalovsky S, Whitby RD. Bio-effects of non-ionizing electromagnetic fields in context of cancer therapy. Frontiers in Bioscience. 2014; 6: 175–184. 24389151 10.2741/e700

[44] Focke F, Schuermann D, Kuster N, Schar P. DNA fragmentation in human fibroblasts under extremely low frequency electromagnetic field exposure. Mutation Res. 2010; 683: 74–83. 10.1016/j.mrfmmm.2009.10.012 19896957

[45] Wang T, Nie Y, Zhao S, Han Y, Du Y, Hou Y. Involvement of midkine expression in the inhibitory effects of low-frequency magnetic fields on cancer cells. Bioelectromagnetics. 2011; 32: 443–452. 10.1002/bem.20654 21360556

[46] Conti P, Giagant GE, Cifone MG, Alesse E, Fieschi C, Bologna M, et al Mitogen dose-dependent effect of weak pulsed electromagnetic field on lymphocyte blastogenesis. FEBS. 1986; 199: 130–134 3082675 10.1016/0014-5793(86)81238-8

[47] Yamaguchi S, Ogiue-Ikeda M, Sekino M, Ueno S. Effects of pulsed magnetic stimulation on tumor development and immune functions in mice. Bioelectromagnetics. 2006; 27: 64–72. 16304693 10.1002/bem.20177

[48] Nie Y, Du L, Mou Y, Xu Z, Weng L, Du Y, et al Effect of low frequency magnetic fields on melanoma: tumour inhibition and immune modulation. BMC Cancer. 2013; 13: 582–593. 10.1186/1471-2407-13-582 24314291 PMC4029221

[49] Novikov VV, Novikov GV, Fesenko EE. Effect of weak combined static and extremely low-frequency alternating magnetic fields on tumor growth in mice inoculated with the Ehrlich ascites carcinoma. Bioelectromagnetics. 2009; 30:343–351. 10.1002/bem.20487 19267367

[50] Pall ML. Electromagnetic fields act via activation of voltage-gated calcium channels to produce beneficial or adverse effects. J Cell Mol Med. 2013; 17: 958–965. 10.1111/jcmm.12088 23802593 PMC3780531

[51] Lisi A, Ledda M, Rosola E, Pozzi D, D’Emilia E, Guiliani L, et al Extremely low frequency electromagnetic field exposure promotes differentiation of pituitary corticotrope-derived AtT20 D16V cells. Biolectromagnetics. 2006; 27: 641–651. 10.1002/bem.20255 16838272

[52] Piacentini R, Ripoli C, Mezzogori D, Azzena GB, Grassi C. Extremely low frequency electromagnetic fields promote in vitro neurogenesis via upregulation of Ca(v)1-channel activity. J Cell Physiol. 2008; 215: 129–139. 17941084 10.1002/jcp.21293

[53] Taylor JT, Zeng XB, Pottle JE, Lee K, Wang AR, Yi SG, et al Calcium signaling and T-type calcium channels in cancer cell cycling. World J Gastroenterol. 2008a; 14: 4984–4991. 18763278 10.3748/wjg.14.4984 PMC2742923

[54] Capiod T (2011) Cell proliferation, calcium influx, and calcium channels. Biochimie 93: 2075–2079. 10.1016/j.biochi.2011.07.015 21802482

[55] Taylor JT, Huang L. Pottle JE, Liu K, Yang Y, Zeng X, et al Selective blockade of T type Ca2+ channels suppresses human breast cancer cell proliferation. Cancer Lett. 2008b; 267:116–124. 10.1016/j.canlet.2008.03.032 18455293

[56] Ohkubo T, Yamazaki J. T-type voltage-activated calcium channel Cav3.1, but not Cav3.2, is involved in the inhibition of proliferation and apoptosis in MCF-7 human breast cancer cells. Int J Oncol. 2012; 41: 267–275. 10.3892/ijo.2012.1422 22469755

[57] Hirooka K, Bertolesi GE, Kelly ME, Denovan-Wright EM, Sun X, Hamid J, et al T-Type calcium channel alpha1G and alpha1H subunits in human retinoblastoma cells and their loss after differentiation. J Neurophysiol. 2002; 88: 196–205. 12091545 10.1152/jn.2002.88.1.196

[58] Iftinca MC. Neuronal T-type calcium channels: what's new? J Med Life. 2011; 4: 126–138. 21776294 PMC3124264

[59] See V, Rajala NK, Spiller DG, White MR. Calcium-dependent regulation of the cell cycle via a novel MAPK-NF-kB pathway in Swiss 3T3 cells. J Cell Biol. 2004; 166: 661–672. 15326199 10.1083/jcb.200402136 PMC2172420

[60] Resende RR, Adhikari A, da Costa JL, Lorencon E, Ladeira MS, Guatimosim S, et al Influence of spontaneous calcium events on cell-cycle progression in embryonal carcinoma and adult stem cells. Biochim Biophys Acta. 2010; 1803: 246–260. 10.1016/j.bbamcr.2009.11.008 19958796

[61] Yam CH, Fung TK, Poon RY. Cyclin A in cell cycle control and cancer. Cell Mol. Life Sci. 2005; 59: 1317–1326. 10.1007/s00018-002-8510-y PMC11337442 12363035

[62] Choi HJ, Fukui M, Zhu BT. Role of cyclin B1/Cdc2 up-regulation in the development of mitotic prometaphase arrest in human breast cancer cells treated with nocodazole. PLOS ONE. 2011; 6: e24312 10.1371/journal.pone.0024312 21918689 PMC3168870

