# Supplementary material for: Inhibition of Cancer Cell Growth by Exposure to a Specific Time-Varying Electromagnetic Field Involves T-Type Calcium Channels
Source: PLoS One. 2015 Apr 14;10(4):e0124136. doi: 10.1371/journal.pone.0124136 (PMC4397079; doi:10.1371/journal.pone.0124136)
Supplement: S1 Fig — (PPTX) [file pone.0124136.s001.pptx]

## Slide 1
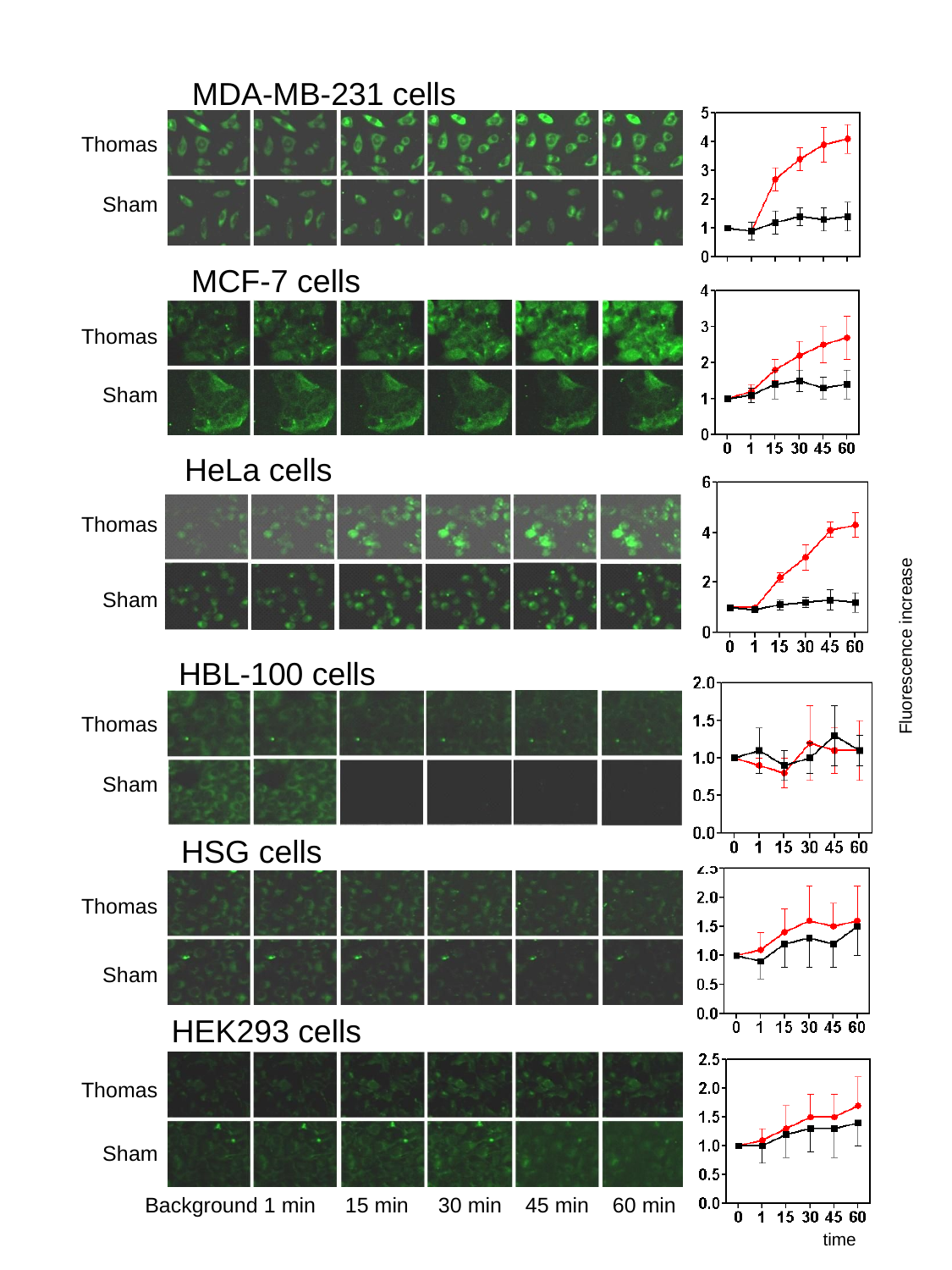

MDA-MB-231 cells
Thomas
Sham
MCF-7 cells
Thomas
Sham
HeLa cells
Thomas
Sham
Fluorescence increase
HBL-100 cells
Thomas
Sham
HSG cells
Thomas
Sham
HEK293 cells
Thomas
Sham
Background 1 min 15 min 30 min 45 min 60 min
time
