# Supplementary material for: Inhibition of Cancer Cell Growth by Exposure to a Specific Time-Varying Electromagnetic Field Involves T-Type Calcium Channels
Source: PLoS One. 2015 Apr 14;10(4):e0124136. doi: 10.1371/journal.pone.0124136 (PMC4397079; doi:10.1371/journal.pone.0124136)
Supplement: S2 Fig — (PPTX) [file pone.0124136.s002.pptx]

## Slide 1
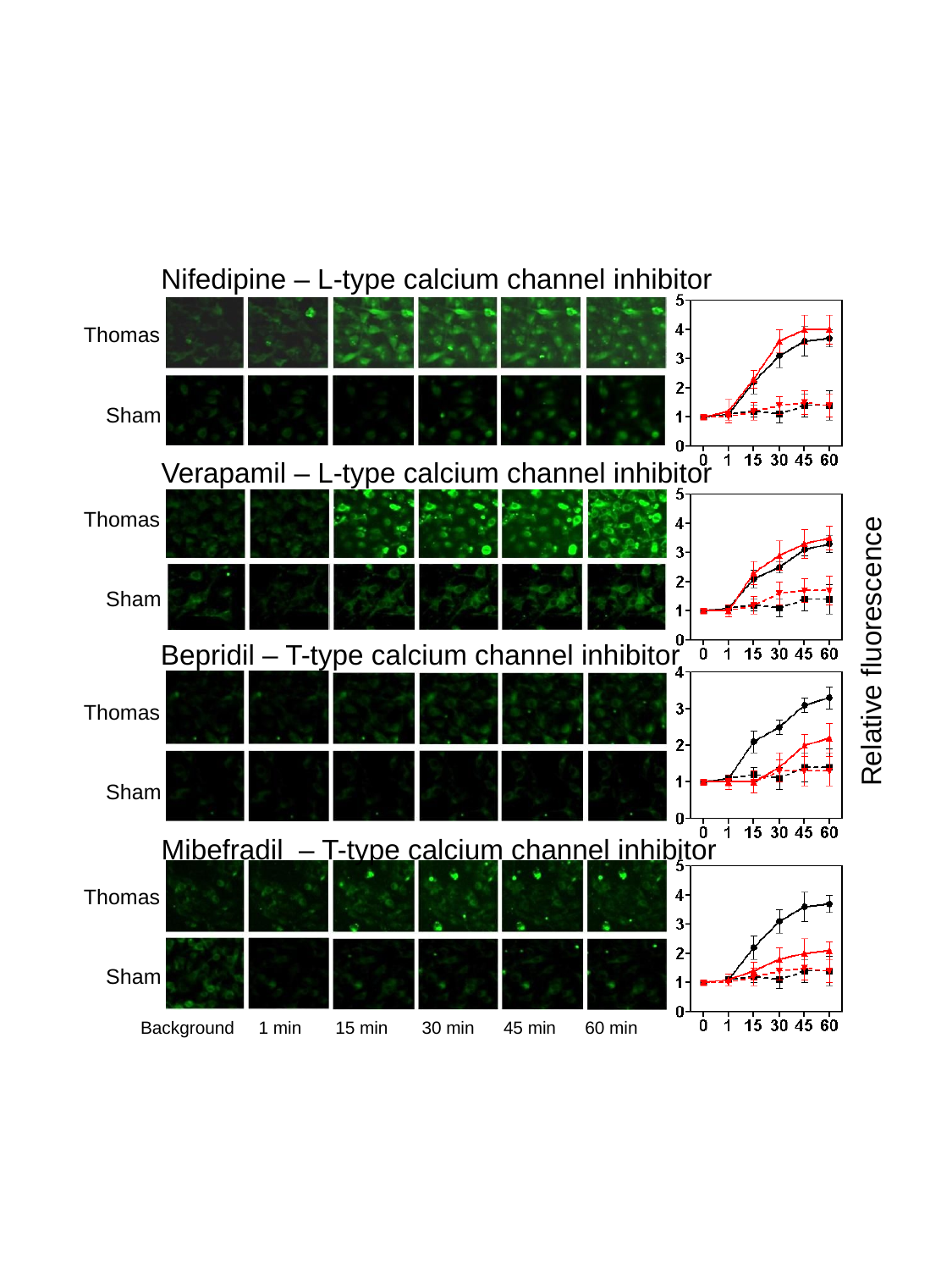

Nifedipine – L-type calcium channel inhibitor
Thomas
Sham
Verapamil – L-type calcium channel inhibitor
Thomas
Sham
Relative fluorescence
Bepridil – T-type calcium channel inhibitor
Thomas
Sham
Mibefradil – T-type calcium channel inhibitor
Thomas
Sham
Background 1 min 15 min 30 min 45 min 60 min
